# The efficacy of oral vitamin D supplements on fusion outcome in patients receiving elective lumbar spinal fusion—a randomized control trial

**DOI:** 10.1186/s12891-022-05948-9

**Published:** 2022-11-18

**Authors:** Ming-Hsien Hu, Yu-Kai Tseng, Yu-Hsuan Chung, Nai-Yuan Wu, Chi-Huan Li, Pei-Yuan Lee

**Affiliations:** 1 Department of Orthopedics, Show Chwan Memorial Hospital, Changhua, Taiwan; 2 Bachelor’s Program of Design and Materials for Medical Equipment and Devices, College of Nursing and Health Sciences, Da-Yeh University, Changhua, Taiwan; 3 College of Medicine, National Chung Hsing University, Taichung, Taiwan; 4 PhD Program in Translational Medicine, College of Life Sciences, National Chung-Hsing University, Taichung, Taiwan; 5 Research Assistant Center, Show Chwan Memorial Hospital, Changhua, Taiwan; 6 Department of Orthopedics, Chang Bing Show Chwan Memorial Hospital, No. 6 Lugong Rd. Lukang Zhen, Changhua, Changhua County Taiwan; 7 Graduate Institute of Biomedical Engineering, National Chung-Hsing University, Taichung, Taiwan

**Keywords:** Vitamin D, Cholecalciferols, Spinal fusion, Fusion, Pseudoarthrosis

## Abstract

**Background:**

Previous studies have reported that vitamin D supplement could improve fracture healing, but evidence regarding the role of vitamin D supplements in spinal fusion was limited. Thus, this study aimed to evaluate the effectiveness of oral vitamin D supplements on fusion outcomes in patients undergoing lumbar spinal fusion.

**Methods:**

This randomized, double-blind, parallel-designed, active-control trial included the patients who planned for elective lumbar spinal fusion. Eligible patients were randomly assigned to receive either daily vitamin D3 (cholecalciferol) 800 IU and daily calcium citrate 600 mg (experimental group) or only daily calcium citrate 600 mg (control group). All supplements were given from postoperative day 1 and lasted for 3 months. Primary outcome was postoperative 1-year fusion rate, and secondary outcomes included time to fusion, Oswestry Disability Index (ODI), and visual analogue scale (VAS) for pain.

**Results:**

Among the included 34 patients (21 in the experimental group and 13 in the control group), baseline 25-hydroxyvitamin D (25[OHVitD) level was 26.7 (10.4) ng/ml. Preoperative prevalence of vitamin D deficiency and insufficiency were 23.5% and 47.1%, respectively. Postoperative 1-year fusion rate was not significantly different between the two groups (95.2% vs. 84.6%, *P* = 0.544). The experimental group had significantly shorter time to fusion (Kaplan–Meier estimated: 169 days vs. 185 days [interquartile range: 88–182 days vs. 176–324 days], log-rank test: *P* = 0.028), lower postoperative 6-month ODI (*P* < 0.001), and lower postoperative 6-month VAS (*P* < 0.001) than the control group. Time to fusion was significantly and negatively correlated with preoperative, postoperative 3-month, and 6-month 25(OH)VitD levels (all *P* < 0.01).

**Conclusion:**

The patient with vitamin D supplements had shorter time to fusion, better spinal function and less pain after elective spinal fusion. Further research is warranted to identify the patients who can benefit the most from vitamin D supplements and the appropriate dose of vitamin D supplements.

**Trial registration:**

ClinicalTrials.gov, NCT05023122. Registered 20 August 2021. Retrospectively registered, http://clinicaltrials.gov/ct2/show/NCT03793530.

**Supplementary Information:**

The online version contains supplementary material available at 10.1186/s12891-022-05948-9.

## Introduction

Spinal fusion is currently the main treatment for various spine pathologies, including degenerative spinal instability, degenerative disc disease, severe spondylolisthesis, spinal stenosis, and spinal deformity [1–3]. The procedure rate of lumbar spinal fusion has increased significantly over the past decade [3–5]. Despite the advancement of surgical techniques, pseudoarthrosis remains one of the most common complications, resulting in poor functional outcomes and increasing risk of reoperation after spinal fusion [6–8]. The process of bony fusion is a dynamic bone remodeling process [9]. A variety of risk factors have been identified to contribute to pseudoarthrosis, for example, osteoporosis, long-term steroid use, and smoking [7, 8, 10, 11]. Notably, one prevalent risk factor is vitamin D deficiency [12, 13].

Vitamin D, an endogenous hormone, plays an important role in the homeostasis of bone remodeling. Vitamin D deficiency can increase bone resorption through reducing calcium absorption and inducing secondary hyperparathyroidism. The increased bone resorption results in increased bone loss and bone turnover, leading to osteoporosis and elevated fracture risk [14].

Vitamin D deficiency has been reported to be associated with more pseudoarthrosis, prolonged time to fusion, and poorer spine function and quality of life after spinal fusion [15–17]. As vitamin D deficiency is correctable with oral supplements, several studies have demonstrated that vitamin D supplements could increase post-fracture bone mineral density (BMD) [18], improve fracture healing [19], and reduce risk of fall and non-vertebral fracture [20].

However, as the review article presented, it lacks high-quality evidence to investigate the role of vitamin D supplements in spinal fusion [21]. Therefore, this randomized controlled trial aimed to evaluate the effectiveness of oral vitamin D supplements on fusion outcomes in patients receiving elective lumbar spinal fusion.

## Methods

### Subjects

This randomized, double-blind, parallel-designed, active-control trial was ethically approved by the Institutional Review Board in Show Chwan Memorial Hospital (No. RA16010) and retrospectively registered on 20 August 2021 (ClinicalTrials.gov: NCT05023122). The included subjects were the patients who aged between 20 and 80 years and planed for elective spine fusion surgery for spinal stenosis or degenerative spondylolisthesis at the study hospital from January 2016 to December 2017. Patients who met one of the following criteria were excluded: (1) spinal instability due to trauma, infection, or malignancy; (2) history of previous spine surgery; (3) hemodialysis; (4) long-term steroid use; (5) history of medical treatments for osteoporosis; (6) postoperative follow-up for less than 12 months; (7) newly-onset compression fracture after study procedure.

### Procedure

We assessed the eligibility at orthopedic outpatient department in Show Chwan Memorial Hospital. For the eligible patients, we discussed with them and if they were willing to participate in this study, they would provide written informed consents and then received operations. All operations were performed by a single spine surgeon using transforaminal lumbar interbody fusion with PEEK cages and autograft. The operated spine levels were from L1 to S1. After surgery, the patients were randomly assigned to either the experimental or control groups using simple randomization method, which is based on a computer generalized sequence of random numbers. The surgeon and patients were unaware of the group assignment. The experimental group received vitamin D3 (cholecalciferol) 800 IU QD and calcium citrate 600 mg QD, whereas only calcium citrate 600 mg QD for the control group. All supplements were centrally prepared with the same appearance (Prince Pharmaceutical Co., Ltd, Taiwan) and were given from postoperative day 1 and lasted for 3 months, and had the same. All patients were followed-up for at least 12 months. We kept recruiting patients until the end of the study period (December 31, 2017); therefore, sample size calculation was not performed, but post-hoc statistical power was analyzed. Because this study was registered retrospectively, all procedures could not be proved to be pre-specified.

### Evaluation and data collection

**Fig. 1:**
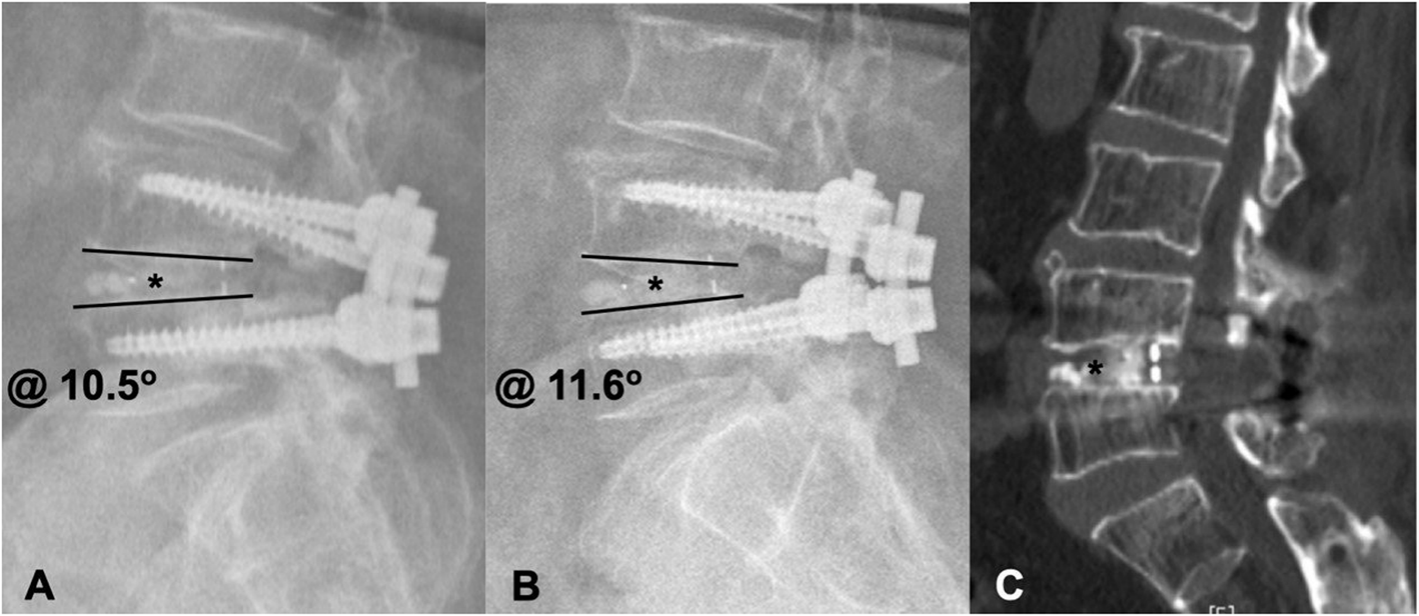
A 75-year-old female received lumbar spinal fusion surgery (L4/5). Lateral flexion (**A**) and extension (**B**) views of lumber spine radiography at postoperative 6-month showed bridging callus formation (labeled by *) and range of motion in L4/5 of less than five degrees (from 10.5 degrees to 11.6 degrees). Computed tomography (**C**) at postoperative 6-month showed callus formation (*) between the fused vertebrae

Fusion status and time to fusion were assessed by radiography and computed tomography (CT) of the lumbar spine [22, 23]. As the example shown in Fig. 1, fusion was defined as the presence of bridging callus without radiolucent line (Fig. 1A, B), spinal range of motion between two fused vertebrae less than 5 degrees (Fig. 1C), and no implant loosening or failure on lumbar spine radiography, [24, 25]. Lumbar spine radiography was performed at postoperative 1-, 2-, 3-, 6, and 12-month visits and lumbar spine CT was followed 6 months postoperatively. All images were interpreted independently by two surgeons who did not involve in this study. All patients were also evaluated for pain with visual analogue scale (VAS), spine function with Oswestry Disability Index (ODI), 25-hydroxyvitamin D (25[OH]VitD) level, and calcium level at preoperative, postoperative 3-month, and 6-month visits. The VAS score ranges from 0 to 10 (0 = least pain, 10 = worst pain). The ODI contains 10 patient-completed questions addressing spinal function. Each question is presented as 6-point Likert scales from 0 to 5 (0 = best outcome, 5 = worst outcome). The overall ODI score ranges from 0 to 100% and a lower score indicates better function [26].

Reference range of serum 25(OH)VitD level is 30 to 60 ng/ml. Vitamin D deficiency and insufficiency are defined as 25(OH)VitD level < 20 ng/ml and 20 to 29.9 ng/ml, respectively [27].

Primary outcome was fusion rate at postoperative 1 year, and secondary outcomes included time to fusion, ODI at postoperative 3 months and 6 months, and VAS at postoperative 3 months and 6 months. Demographic data, smoking status, body mass index (BMI), T-score, BMD, operation data (i.e., operation time, amount of blood loss, and number of the fused vertebrae), and surgical complications (e.g., infection) were recorded.

### Statistical analysis

Continuous variables were presented as mean (standard deviation) and categorical variables as count (percentage). Comparison of the characteristics and outcomes between the experimental and control groups were performed by Mann–Whitney U test and Fisher’s exact test for continuous and categorical variables, respectively. Association between continuous variables were assessed by Pearson’s correlation. Time to fusion was estimated by Kaplan–Meier method and compared by log-rank test. A two-tailed *P* < 0.05 indicated statistical significance. All statistical analyses were performed using IBM SPSS Statistics for Windows, Version 24.0 (Armonk, NY, USA).

## Results

This trial included a total of 34 patients (21 in the experimental group and 13 in the control group) with a mean age of 61 (13.5) year and 24 (70.6%) patients were female. Mean of preoperative 25(OH)VitD level in all patients was 26.7 (10.4) ng/ml and was not significantly different between the experimental and control groups (26.8 vs. 26.4, *P* = 0.607). Eight (23.5%) patient had vitamin D deficiency and 16 (47.1%) had vitamin D insufficiency. As presented in Table 1, the experimental and control groups had similar demographic characteristics, baseline information, preoperative evaluation, and operation data (Table 1).

**Table 1:** Demographic and preoperative characteristics of the patients receiving elective lumbar spinal fusion

	Experimental group (*n* = 21)	Control group (*n* = 13)	*P*
Female^a^	17 (81.0)	7 (53.8)	0.130
Age (years)	64.0 (11.1)	56.1 (15.0)	0.136
Smoking^a^	4 (19.0)	3 (23.1)	1.000
Body mass index (kg/m^2^)	23.9 (3.5)	28.0 (6.9)	0.049
Bone mineral density (g/cm^2^)	0.8 (0.2)	0.8 (0.2)	0.242
T-score	-2.0 (1.3)	-1.2 (1.1)	0.079
Preoperative evaluation			
ODI	22.3 (9.8)	20.9 (6.3)	0.558
VAS	7.2 (1.9)	6.2 (1.6)	0.135
25(OH)VitD (ng/ml)	26.8 (8.8)	26.4 (12.9)	0.607
Calcium (mg/dl)	8.9 (0.5)	8.7 (0.7)	0.424
Operation data			
Blood Loss (ml)	300.5 (290.3)	390.0 (288.2)	0.206
Operation time (min)	166.2 (51.2)	195.8 (57.6)	0.106
Number of fused vertebrae	2.3 (0.5)	2.8 (1.0)	0.248

*Abbreviation: ODI* Oswestry Disability Index, *VAS* Visual analogue scale

^a^ Data were presented as mean (standard deviation) or count (percentage)

**Fig. 2:**
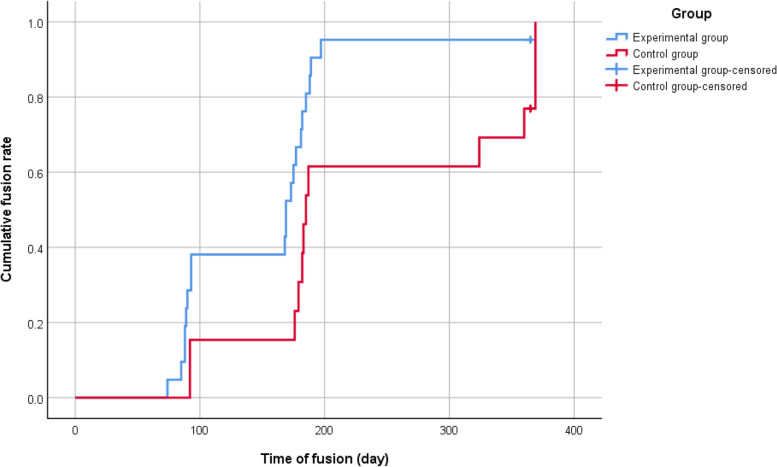
Kaplan–Meier analysis showed a significantly shorter time to fusion in the experimental group than in the control group (169 days vs. 185 days [interquartile range: 88–182 days vs. 176–324 days], *P* = 0.028)

All patients completed this study. No patients dropped out during the study or were excluded from the study after surgery. The primary analysis was intention-to-treat and involved all patients who were randomly assigned (Supplementary Fig. 1). Table 2 presented the primary and secondary outcomes of this trial. Postoperatively, 1-year fusion rate was not significantly different between the experimental and control groups (95.2% vs. 84.6%, *P* = 0.544). The experimental group had significantly shorter time to fusion than the control group (169 days vs. 185 days [interquartile range: 88–182 days vs. 176–324 days], *P* = 0.028, Fig. 2). During the entire study period, no deep infection was observed and only two patients in the control group experienced postoperative infection, which was resolved by antibiotics (Table 2).

**Table 2:** Postoperative outcomes of the patients receiving elective lumbar spinal fusion

	Experimental group (*n* = 21)	Control group (*n* = 13)	*P*
Fusion^a^	20 (95.2)	11 (84.6)	0.544
Infection^a^	0 (0)	2 (15.4)	0.139
Postoperative 3-month evaluation			
ODI	7.0 (5.4)	11.0 (4.8)	0.007
VAS	2.8 (1.5)	3.8 (1.2)	0.078
25(OH)VitD (ng/ml)	31.9 (10.0)	27.9 (13.8)	0.184
Calcium (mg/dl)	9.2 (0.4)	9.1 (0.4)	0.319
Postoperative 6-month evaluation			
ODI	2.3 (1.6)	8.4 (3.9)	< 0.001
VAS	1.5 (1.0)	3.2 (1.0)	< 0.001
25(OH)VitD (ng/ml)	31.6 (8.2)	27.4 (12.9)	0.092
Calcium (mg/dl)	9.0 (0.4)	9.0 (0.3)	0.887

*Abbreviation: ODI* Oswestry Disability Index, *VAS* Visual analogue scale

^a^ Data were presented as mean (standard deviation) or count (percentage)

The experimental group had significant lower ODI scores than the control group at postoperative 3 month (7.0 vs. 11.0, *P* = 0.007) and 6 month (2.3 vs. 84, *P* < 0.001). VAS scores were similar between the two groups at postoperative 3 month, but were significantly lower in the experimental group than in the control group at postoperative 6 month (1.5 vs. 3.2, *P* < 0.001). 25(OH)VitD and calcium levels were comparable between the two groups after surgery. No patient reported adverse effects due to oral vitamin D supplements (Table 2). No patients had signs of vitamin D over-supplementation, such as hypercalcemia (serum calcium > 10.5 mg/dl) or had any symptoms suggesting vitamin D intoxication, such as weakness, fatigue, anorexia, and bone pains.

For patients with bony fusion after spinal fusion surgery, Table 3 presented the association of fusion outcomes with 25(OH)VitD level, calcium level, and preoperative characteristics. In the 31 patients with fusion at postoperative 1 year, time to fusion was significantly and negatively correlated with preoperative (*r* = -0.582), postoperative 3-month (*r* = -0.489), and 6-month (*r* = -0.496) 25(OH)VitD levels (all *P* < 0.01), whereas preoperative calcium level and preoperative characteristics (i.e., BMI, BMD, operation time, and number of fused vertebrae) had no effect on the time to fusion (Table 3). The negative correlation of time to fusion with preoperative 25(OH)VitD level and with postoperative 6-month 25(OH)VitD level were also observed in the individual experimental and control group. The experimental group had greater percentage change in the correlation coefficient of time to fusion and 25(OH)VitD level from preoperative to postoperative 6-month (*r*, -0.543 to -0.285, 47.5%) than the control group (*r*, -0.769 to -0.601, 21.9%). Post-hoc power analysis revealed the statistical power of the study to be 81% for detecting significant difference in time to fusion between the two groups.

**Table 3:** Association of fusion outcomes with vitamin D level, calcium level, and preoperative characteristics in patients with bony fusion after spinal fusion surgery

	Time to fusion (day)	ODI, postop 3 m	ODI, postop 6 m	VAS, postop 3 m	VAS, postop 6 m
BMI (kg/m^2^)	0.166	0.142	0.393^*^	0.061	0.269
BMD (g/cm2)	0.022	0.182	0.143	-0.028	0.169
Number of fused vertebrae	0.126	0.159	0.096	-0.003	-0.079
Operation time (min)	0.011	-0.043	-0.082	-0.035	-0.087
25(OH)VitD, preop (ng/ml)	-0.582^**^	0.065	0.018	-0.050	0.031
25(OH)VitD, postop 3 m (ng/ml)	-0.489^**^	-0.117	-0.170	-0.260	-0.204
25(OH)VitD, postop 6 m (ng/ml)	-0.496^**^	-0.176	-0.182	-0.252	-0.242
Calcium, preop (mg/dl)	0.063	-0.144	-0.07	-0.013	-0.019

*Abbreviation: BMI* Body mass index, *BMD* Bone mineral density, *ODI* Oswestry Disability Index, *VAS* Visual analogue scale, *preop* preoperative; postop, postoperative, *3 m* three months, *6 m* six months

^*^P < 0.05

^**^P < 0.01

## Discussion

As vitamin D deficiency has been identified as a risk factor of pseudoarthrosis after orthopedic surgery [12, 13], vitamin D supplements might improve fusion outcomes in patients receiving elective spinal fusion. Although this study did not find significant difference in 1-year fusion rate between the experimental and control groups, the patients with vitamin D supplements had significantly shorter time to fusion, better spinal function, and less pain after elective spinal fusion than those without vitamin D supplements. To the best of our knowledge, this is the first randomized controlled trial to evaluate the effectiveness of vitamin D supplements on fusion outcomes in patients receiving elective spinal fusion.

Our results on the effect of vitamin D supplements on fusion rate and time to fusion were consistent with findings from previous observational studies. Ravindra et al. reported that low 25(OH)VitD level was significantly associated with longer time to fusion and was an independent risk factor of union after spinal fusion surgery [15]. An animal model with rats after spinal fusion surgery revealed a dose dependent relationship between vitamin D supplements and fusion rate [28]. Several studies have also observed that higher 25(OH)VitD level was associated with the improvement of pain and function [21]. Waikakul et al. reported that nine patients with failed back surgery syndrome had significantly improved pain and back function after vitamin D2 and vitamin D3 supplements for 6 months [29]. Xu et al. reported significantly worse VAS and ODI outcomes in vitamin D deficient patients after elective lumbar spine surgery [30]. However, In an observational study including 150 patients undergoing lumbar spinal fusion surgery, both preoperative and postoperative 25(OH)VitD level were not found to be significantly associated with pseudoarthrosis and VAS at postoperative 1 year [31]. Further research is necessary to revisit these controversial results.

The prevalence of vitamin D deficiency and insufficiency in this study were respectively 23.5% and 47.1%, which were comparable with previous reports. A cross-sectional study by Ravindra et al. observed that 30.0% and 38.9% of patients had vitamin D deficiency and insufficiency, respectively [13]. Stoker et al. reported that prevalence of vitamin D deficiency and insufficiency were 27% and 57%, respectively [12].

The experimental and control groups in this study had comparable preoperative 25(OH)VitD levels and fusion rates. Meanwhile, multivariate analysis could not be conducted due to limited sample size. Therefore, this study could not figure out the effect of preoperative vitamin D deficiency on fusion rate. However, the present study found that preoperative 25(OH)VitD level negatively correlated with time to fusion. Notably, the percentage change in the correlation of time to fusion and 25(OH)VitD level from preoperative to postoperative 6 months (*r*, -0.543 to -0.285, 47.5%) was greater in the experimental group than that in the control group. The results might suggest that the negative impact of lower baseline vitamin D deficiency on time to fusion could be attenuated by postoperative vitamin D supplements. However, in the present study, the 25(OH)VitD level in the experimental group only slightly increased from 26.8 ng/ml preoperatively to 31.9 ng/ml at postoperative 3 month and to 31.6 ng/ml at postoperative 6 months. By comparison, in the study by Doetch et al., the 25(OH)VitD level significantly increased from 40 nmol/l (16 ng/ml) to 72 nmol/l (28.9 ng/ml) after supplementation of vitamin D3 800 IU for 3 months [18]. The relatively less increase in 25(OH)VitD level in our patients might be attributable to higher preoperative 25(OH)VitD level. Several studies have demonstrated the inverse relationship of baseline 25(OH)VitD level with response to vitamin D supplements [32, 33]. Therefore, if we consider to provide vitamin D supplements before spine surgery by a routine approach, it need to identify the patients who can benefit the most from vitamin D supplements and to determine the appropriate dosage of vitamin D supplements [21].

In this study, the duration of vitamin D supplement was 3 months because previous studies have demonstrated that vitamin D deficiency can typically be corrected in 6 to 12 weeks, leading to stabilization of bone turnover [21]. Furthermore, the design of 3-month vitamin D supplement has been used in several randomized controlled trials, for example, in one trial of the effect of vitamin D supplement on musculoskeletal function [34] and in the other one of the effect of vitamin D supplement on healing process of osteoporotic fracture [18].

There are some inherent limitations in this study. The first came from the limited sample size. Therefore, simple randomization method used in this study resulted in relatively unequal group sizes. Post-hoc power analysis revealed the statistical power of the study to be 81% for detecting significant difference in time to fusion between the two groups. However, large-scale clinical trials with appropriate randomization (e.g., block randomization method) are warranted and our results could serve as a base for future studies. Secondly, time to fusion was determined by frequent radiography at postoperative 1-, 2-, 3-, 6, and 12-month visits. It might be less precise to detect the real time to fusion. Although radiography is the most common imaging modality in orthopedic practice, it is necessary to assess the time to fusion in a more accurate manner. Thirdly, we did not consider the patient adherence to the supplements given in this study, the amount of vitamin D in diet, and degree of sunlight exposure [35]. The difference in fusion outcomes between the experimental and control groups might be reduced by poor adherence of vitamin D supplement. Although the results might be interfered, this study could reflect the real-world situation. Future studies need to consider these factors. Fourth, this trial was not prospectively registered on ClinicalTrials.gov. We could not prove that all procedures were pre-specified. There was the possibility of failure of randomization of all covariates. Finally, the comparisons of the two groups were not adjusted for demographic and preoperative characteristics by multivariate analysis because of the limited sample size. Bony fusion has been identified to be associated with age, osteoporosis, and vitamin D deficiency, and meanwhile 25(OH)VitD level also correlates with age and osteoporosis [14, 18]. Therefore, the role of vitamin D in moderating and mediating the fusion process deserves further investigation.

## Conclusion

This randomized control trial revealed that the patients with vitamin D supplements had shorter time to fusion, better spinal function, and less pain after elective spinal fusion surgery. Postoperative vitamin D supplements might attenuate the negative impact of lower 25(OH)VitD level on time to fusion. Further research is needed to identify the patients who can benefit the most from vitamin D supplements and to determine the appropriate dose of vitamin D supplements while we consider to provide vitamin D before spine surgery in a routine manner.

## Supplementary Information

**Additional file 1: Supplementary Figure 1.** Study flow.

## Data Availability

The data that support the findings of this study are available from the corresponding author upon reasonable request.

## Abbreviations

25[OH]VitD: 25-Hydroxyvitamin D
BMD: Bone mineral density
VAS: Visual analogue scale
ODI: Oswestry Disability Index
BMI: Body mass index
