# Supplementary figures and images for: The efficacy of oral vitamin D supplements on fusion outcome in patients receiving elective lumbar spinal fusion—a randomized control trial
Source: BMC Musculoskelet Disord. 2022 Nov 18;23:996. doi: 10.1186/s12891-022-05948-9 (PMC9673414; doi:10.1186/s12891-022-05948-9)

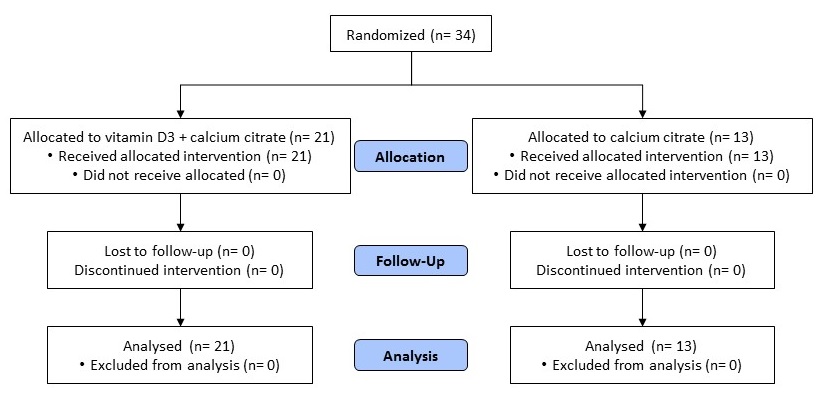

Supplement: Supplementary file 1 — Additional file 1: Supplementary Figure 1. Study flow. [file 12891_2022_5948_MOESM1_ESM.jpg]
